# Complementary use of the Ecosystem Service Concept and Multi-criteria Decision Analysis in Water Management

**DOI:** 10.1007/s00267-021-01501-x

**Published:** 2021-07-26

**Authors:** Mika Marttunen, Jyri Mustajoki, Virpi Lehtoranta, Heli Saarikoski

**Affiliations:** Finnish Environment Institute, SYKE, Latokartanonkaari 11, 00790 Helsinki, Finland

**Keywords:** Ecosystem service, Multi-criteria decision analysis, Stakeholder, Water, Management

## Abstract

The ecosystem service (ES) concept has increasingly been applied in environmental planning, while there are several decades of experience in applying multi-criteria decision analysis (MCDA) in complex planning situations. The aim of this article is to assess how the ES concept has been used in water management projects together with MCDA and to examine the experiences gained and make recommendations to overcome any identified challenges. Our conclusions are based on a systematic analysis of 23 articles that were selected among 206 articles focused on water-related studies using, for example, the terms multi-criteria and ecosystem services in the title, abstract or keywords. Here, we explore (i) at what level of detail ESs are included in the decision hierarchy, (ii) the pros and cons of the complementary use of the two approaches, and (iii) how the potential challenges related to the use of MCDA, such as the large number of criteria, double-counting, or assigning criteria weights, are addressed in the selected cases. The results reveal large differences between the case studies. It is shown that only a few case studies used ES categories to classify criteria in the decision hierarchy, that these cases included different numbers of ES criteria and non-ES criteria, and that most case studies elicited stakeholder preferences in MCDA. Although the paper focuses on water management projects, the conclusions regarding the advantages and pitfalls of the complementary use of the methods, as well as our recommendations, are also applicable to other environmental management contexts.

## Introduction

The concept of the services and benefits provided by nature and ecosystems has been developed since the 1970s (Brown et al. 2007; Gómez-Baggethun et al. 2010). Following the stabilization of the terminology into the form of ‘ecosystem services’ (ES) (Costanza et al. 1997; Daily 1997), it has become a widely known and mainstream paradigm with established practices for analyzing the benefits that people obtain from ecosystems (Chen et al. 2020). Since then, the ES concept has increasingly been applied in land use planning and water management (e.g., von Haaren and Albert 2011; Cook and Spray 2012; Grizzetti et al. 2016).

Water management decision-making situations usually involve trade-offs between competing ESs. Hence, there is growing interest in multi-criteria decision analysis (MCDA) methods, which are suited to assessing complex decision-making situations with multiple and mutually exclusive objectives (Belton and Stewart 2002; Keisler and Linkov 2014). MCDA methods have several benefits in ES assessment and valuation, such as a structured process of value-focused thinking, integration of subjective views into the evaluation, and non-monetary valuation (Chan et al. 2012; Keune and Dendoncker 2013; Kenter et al. 2016; Saarikoski et al. 2016).

The use of MCDA in environmental applications has considerably increased in number and diversity during the last decade (Huang et al. 2011; Keisler and Linkov 2014; Cegan et al. 2017). There is also a large body of literature on MCDA in water management planning (e.g., Hajkowicz and Collins 2007), and an emerging body of literature in which MCDA methods have been used to assess and value aquatic ESs (e.g., Borsuk et al. 2019). The impacts of water management projects are typically significant and far-reaching, affecting ESs in many ways (Cook and Spray 2012). Identification of the most feasible and sustainable alternatives requires their systematic evaluation. However, no extensive research has been conducted on water management case studies using MCDA jointly with the ES concept.

In this paper, we assess how the ES concept has been used in water management projects together with MCDA, and what are the advantages and pitfalls of the complementary use of ES and MCDA. The aim is to provide new insights and good practices for developing the complementary use of the methods. We draw conclusions based on the analysis of 24 cases in 23 articles identified to be the most relevant after the screening of 206 articles. Our main research questions are as follows:

- How and at what level have the approaches been interconnected?
- How has the ES concept been used in MCDA and the decision hierarchy?
- What have been the roles of stakeholders in the process and how have different subjective views of the stakeholders been dealt with in the analyses?
- What are the advantages and pitfalls of the complementary use of ES and MCDA?

On the basis of this, we identify good practices and make suggestions for the complementary use of the approaches.

This paper is structured as follows. First, we introduce the ES concept and MCDA, and how they can be applied in a complementary way. This part also presents our research questions and how we conducted the literature review. After that, we present the results of the literature review and the more detailed case analyses. Next, we discuss the advantages and pitfalls of the complementary use of the ES concept and MCDA in water management and present recommendations regarding their complementary use. Finally, we summarize major findings in Conclusions.

## Material and methods

### Approaches under Study

#### Ecosystem service concept

In this paper, we use the term “ES concept” so that besides the idea of assessing the services and benefits provided by nature, it also covers the use of different systems for classifying ESs as well as integrated assessment and valuation processes of ESs. The ES concept as such is not an explicit decision support tool, but rather an awareness raising tool and a common framework for integrating different perspectives and approaches in environmental management (Ainscough et al. 2019). In water management, the ES concept can increase stakeholders’ awareness of the significance of aquatic habitats and encourage them to promote policies that protect freshwater ecosystems together with people’s well-being (Liquete et al. 2016).

There are various purposes of use for the ES concept, and also various ways to implement the process in practice (e.g., Torres et al. 2021). For example, Pendleton et al. (2015) identify the following generic phases for the process: (i) identification of the needs of the case and general scoping, (ii) refinement of the scope and (iii) choice of methods, tools and means for quantifying the assessment. In practice, it is very important to plan the implementation of an ES according to its purpose of use (Heink and Jax 2019).

An influential attempt to create an ES typology was that by the Millennium Ecosystem Assessment (MEA 2005), which classified ESs as supporting, regulating, provisioning, and cultural services. The classification was adopted by the Economics of Ecosystem and Biodiversity (TEEB) framework, in which supporting services were broadened to also include habitat services (TEEB 2008). However, the ES categories are not operable as such in either of these, because they do not distinguish between intermediate ecosystem processes and the services that are directly consumed or enjoyed by people (Boyd and Banzhaf 2007; Fisher and Turner 2008). For instance, if we calculate the value of the regulating service nitrogen removal on the basis of the value of clean drinking water, and sum it up with the value of the provisioning service drinking water, we double-count the contribution of the nitrogen removal service (Saarikoski et al. 2016).

To address double-counting, Boyd and Banzhaf (2007) introduced the notion of final ecosystem goods and services, defined as “components of nature, directly enjoyed, consumed, or used to yield human well-being.” It has been adopted, for example, by the latest version of Common International Classification of Ecosystem Services (CICES V5.1; see www.cices.eu) and the National Ecosystem Services Classification System (NESCS Plus) developed by the U.S. Environmental Protection Agency (Newcomer-Johnson et al. 2020). CICES defines final ecosystem services as the contributions that ecosystems make to human well-being.

In CICES, ESs are classified into provisioning services, regulation and maintenance services, and cultural services, and these are in turn divided into divisions describing the main types of output or process. These are further split into groups based on biological, physical or cultural type or process, and the lowest level is the class, which provides a detailed classification into biological or material outputs and bio-physical and cultural processes. For example, the class “flood protection” belongs to the group of “liquid flows”, which further belongs to the division of “mediation of flows” under the section “regulation and maintenance services”.

Another problem of ES classification systems is that they do not capture all the socioeconomic aspects that are relevant in environmental management and policy-making situations (e.g., Mustajoki et al. 2020, Flood et al. 2020). For example, Proctor and Drechsler (2006) covered social and economic criteria along with ecosystem services criteria in their multicriteria evaluation of recreational opportunities, and Saarikoski et al. (2019) found that jobs and regional economy criteria were needed to complement the assessment of peatland ecosystem services. Yet, another critique toward the ES classification systems is that many services fit into more than one of the four categories. For example, in many countries, food is both a provisioning service and a cultural service (Chan et al. 2012).

#### Multi-criteria decision analysis

Multi-criteria decision analysis (MCDA) is a systematic approach in which the problem is structured into a model that combines objective measurement data on the criteria-wise performances of the alternatives with subjective value judgments about the trade-offs between the criteria (see, Belton and Stewart 2002). It is a so-called non-monetary approach, and it is an alternative to monetary approaches such as cost–benefit analysis (CBA, e.g., Boardman et al. 2017). Non-monetary approaches have been considered useful in ES valuations for several reasons (see, Saarikoski et al. 2016). For example, they enable the multi-dimensional nature of human well-being to be demonstrated, with monetary value being just one aspect of importance amongst others, such as symbolic, cultural, ecological, and spiritual aspects (Chan et al. 2012).

Typically, MCDA process consists of the following phases: (i) structuring of the problem including the identification of the objectives, criteria and measures for them, construction of a decision hierarchy based on all these as well as generation of alternatives, (ii) evaluation of the impacts of the alternatives and creation of a consequence table, (iii) eliciations of opinions and preferences of stakeholders regarding the importance of the objective and assigning the criteria weights, (iv) calculation of the overall priority for alternatives using, for example, Excel or MCDA software, (v) analysis of the results, including sensitivity analysis and recommendations.

The outcome of MCDA is the ranking of alternatives or performance scores of the alternatives, reflecting their suitability to the evaluator. Nowadays, there are several different methods and techniques within MCDA (Zopounidis and Pardalos 2010; Gregory et al. 2012), as well as various tools and software programs to support them (Weistroffer et al. 2005; Janssen and Van Herwijnen 2006; Mustajoki and Marttunen 2017). The methods have different theoretical foundations, such as value functions, optimization algorithms, goal aspiration, and outranking, or a combination of these. They apply different procedures for scoring, weighting, and aggregation (Belton and Stewart 2002). For example, in the widely used Multi-Attribute Value Theory (MAVT, Keeney and Raiffa 1976), weights are elicited from each decision maker to act as scaling factors that determine the relative added value for the decision maker associated with the impact range defined for each criterion (e.g., Eisenführ et al. 2010). Another approach is the Analytic Hierarchy Process (AHP, Saaty 1980), which applies pairwise comparisons of criterion importance to elicit the weights.

MCDA is nowadays a well-established approach (Greco et al. 2016). Thus, there has been much research related to it and there is a good understanding of its strengths and weaknesses. The main challenges reported in the use of MCDA are related to designing the hierarchy in such a way that it adequately, but not in too much detail, describes the decision situation to be examined, and to determining the weights of the criteria. The latter is associated with various biases and sources of error (e.g., Montibeller and von Winterfeld 2015, Marttunen et al. 2018).

**Fig. 1:**
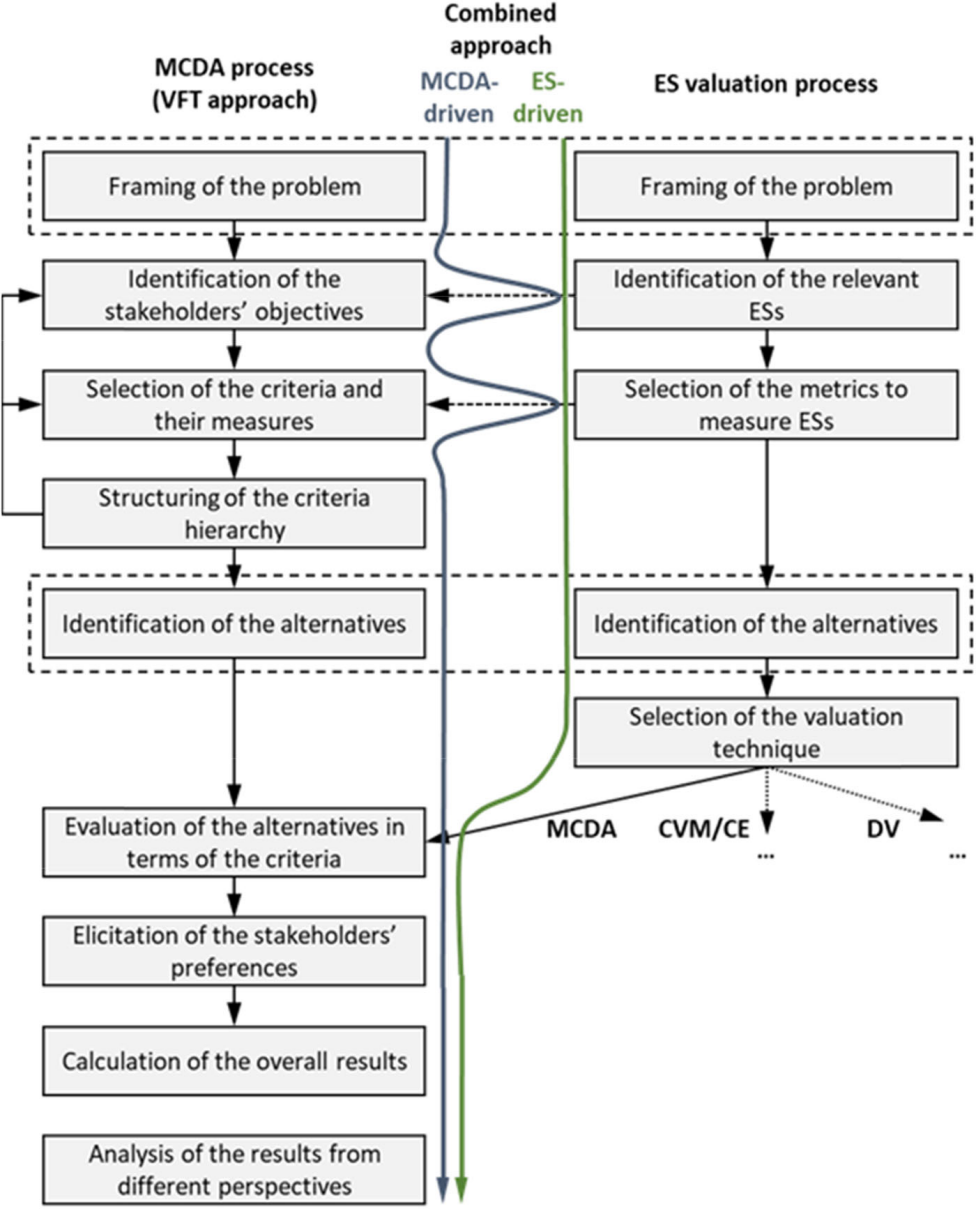
Generic phases of the MCDA process (based on value-focused thinking) and the ES valuation process, and examples of combining of these approaches from the viewpoints of both MCDA and ES. Dashed rectangles mean identical phases

MCDA applications can differ greatly depending on their role in the planning process (e.g., comparison of alternatives only or also providing a framework for the whole planning process) and the role of stakeholders in the process (e.g., Marttunen et al. 2015). In practice, MCDA can be applied in various ways, and in this paper, for clarity, we focus on an MCDA process that builds on a structured process of value-focused thinking (VFT; Keeney 1992; Fig. 1). In many recent MCDA applications, the main aim has not simply been to make a choice between the alternatives but to use the systematic MCDA framework to explore objectives and alternatives, facilitate communication, enhance social learning, and support consensus building (e.g., Antunes et al. 2011; Bana e Costa et al. 2004; Marttunen et al. 2015).

One of the strengths of MCDA is its ability to support multi-stakeholder processes and bring subjective views into the evaluation. There are established procedures for engaging stakeholders and for eliciting their opinions/preferences in a structured way (Borsuk et al. 2019) either on individual basis or in a deliberative fashion. In deliberative MCDA processes, joint weights are determined through group discussion. Deliberated group values can be better informed than group values, and deliberation supports learning and reflection on initial interests and preferences (Raymond et al. 2014). However, using consensual set of weights can be at odds with the notion of value pluralism (Garmendia and Gamboa 2012), and using joint set of values can be challenging in stakeholder processes addressing contentious environmental management problems (Saarikoski et al. 2016).

In a value-focused based MCDA, stakeholders are also involved throughout the process, for example in setting objectives and developing alternative options (e.g., Marttunen et al. 2015). MCDA can additionally be used to combine and structure various stakes and incommensurable diversity related to decision criteria, information, opinions about the impacts, and preferences (see Keune and Dendoncker 2013). MCDA can also act as a link between the ES concept and policy making by providing a means to structure the problem and the preferences of the stakeholders into a form that is applicable to support decision making (Langemeyer et al. 2016).

#### Complementary use of MCDA and the ES concept

MCDA and the ES concept can be applied in various ways to complement each other. Figure 1 presents the generic phases of both MCDA and the ES-based valuation process, and two examples of combining these approaches in a complementary way. The first example is an MCDA-driven process (blue line), building on the methodological foundations of the MCDA approach (see Section 2.1.2), which are then applied to the substance of the ESs. In this, the process mainly follows the basic MCDA process, but in the phases of the identification of objectives and selection of the criteria, it steps out to the ES process to utilize some structured classification system of ESs (e.g., CICES). The second, opposite example is an ES-driven process (green line), which at first follows the substance-based analysis of ESs and moves on to utilize the methodological support provided by MCDA in the valuation of ESs in the later stages of the process. One should note that here, MCDA is just one approach among the possible valuation approaches including, for example, deliberative valuation (; see, Proctor and Drechsler 2006; Pascual et al. 2017) and economic valuation methods, such as the contingent valuation method (CVM) or choice experiments (CE) (see, e.g., Saarikoski et al. 2016).

The main conceptual difference between MCDA- and ES-driven approaches is that the MCDA-driven process follows the principles and phases of MCDA, which is applied to the context of ESs, whereas the ES-driven process starts from the context of ESs, and after the structuring phase, the MCDA process is followed. Another a more functional difference is that MCDA starts from scratch and the criteria are added on the basis of the stakeholders’ values (bottom-up), whereas in the ES-driven process, all the possible criteria (i.e., the ESs) are the starting point for the structuring phase of the problem, of which only the relevant ones are selected for the analysis (top-down). At best, the process integrates the advantages of both approaches.

### Literature Review

We searched for articles from the Web of Science Core Collection (https://www.webofknowledge.com). Our search string consisted of three components. The first component (#1) reflected the focus on the aquatic environment (water or lake or river or stream or watershed or catchment or groundwater or storm). The second term (#2) was “ecosystem service*” and the third component (#3) was related to multi-criteria assessment (multi criteria* or multicriteria* or multiple criteria*). The outputs were combined through Boolean operators using the command: #1 AND #2 AND #3. Search results were sorted by relevance. The screening process was carried out separately by two authors. Titles and abstracts were first screened, and full texts were then considered. Publications were selected that had applied MCDA in a freshwater case study environment. Thus, in this phase, for example, marine cases were excluded. Decisions regarding uncertain papers were discussed with another reviewer. Only publications written in English and available in full-text form were included for the data screening phase.

**Fig. 2:**
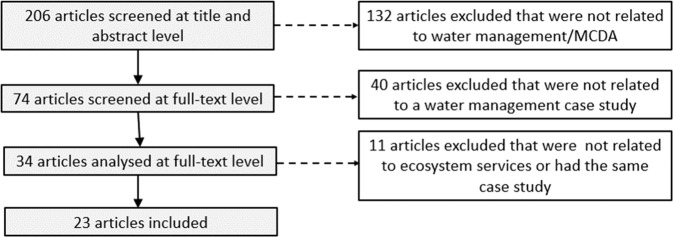
The process for selecting the articles

The study selection process is presented in Fig. 2. In total, 206 articles were identified. Their relevance was assessed based on the title and abstract. A large number of articles (172) were excluded in the first two phases of the screening because they were not real water management cases, as “water” played only minor role in the articles or MCDA was not applied. The remaining 34 articles were selected for closer examination. A further 10 articles were excluded because they did not address ESs. In addition, one article, Mavrommati et al. (2017), was removed, as Borsuk et al. (2019) describe the same case. The remaining 23 articles formed the basis for the final analysis. The following data were extracted, categorized, and stored in an Excel file (see Supplementary Material):

- General publication information (e.g., authors, year, country, journal)
- Type of article (MCDA or ES-driven, or mixed approach)
- Decision situation (e.g., generic example, strategic analysis, or comparison of alternatives)
- Stakeholder involvement in different phases of the process (structuring of the problem, construction of the decision hierarchy, weighting of the criteria, analysis of the results)
- Characteristics of the decision hierarchy (how ES were included in the hierarchy, the total number of criteria, and their number in various ES categories)
- The pros and cons of the applied approach presented in the article

It should be noted that it was not clear-cut which articles could be classified as water management cases and which could not. Among the selected articles were those mainly related, for example, to forestry or agriculture, but which contained a water element. Furthermore, agricultural cases mentioning the word “catchment” were included in the analysis. Although our review cannot be claimed as comprehensive, it covers a good number of articles documenting the use of MCDA methods in ES assessment and valuation.

## Results

### Description and Grouping of the Articles

The analysis is based on a careful review of the 23 articles, using a summary table and a set of criteria to organize the findings (see further details section 2.3). MCDA was applied in the evaluation of sites/alternatives in 22 articles. In one article, multi-criteria analysis was limited to the use of the Delphi method to assess the importance of criteria (Canada and Mariottoni 2016). The most common multi-criteria methods were MAVT/MAUT (10 cases), another generic MCDA method (7 cases), and AHP (4 cases). Other applied method was ELECTRE (1 case). One article (Johnston et al. 2013) reported several cases where the method varied case by case. For detailed information, see Online Resources 2.

The cases cover a wide range of water management sectors (Online Resources 1). The following topics were the most common: river restoration and wetlands (11 articles) and land-use management (8 articles). The rest of the articles dealt with the agricultural water load, watershed protection, water scarcity, urban stormwaters, and green infrastructure, among other topics. Most (14/23) of the selected articles were published in 2015 or later. Of the articles, 22 described a case study and one a summary of seven cases (Johnston et al. 2013). Most (12/23) cases had been conducted at the regional level, nine at the local level, and only one had a national dimension (Odgaard et al. 2017). In the cases presented by Johnston et al. (2013), the perspectives varied from local to regional.

### Decision Hierarchy: how is the ES Concept used in MCDA?

We classified the cases into seven classes (A–G) according to how tightly the ES concept and the ES classification systems were linked to MCDA (Table 1). In the table, the level of linkage decreases with successive class letters from the strongest (class A) to the weakest (class G). One should, however, note that the order of the classes, especially in the middle, is somewhat blurred. For example, which of classes C (in which ES criteria are explicitly identified) or D (in which ES is mentioned as an overarching goal of the analysis) can be judged to be more tightly linked to the ES concept strongly depends on the characteristics of the case. Nevertheless, Table 1 demonstrates large differences in the inclusion of the ES concept in MCDA. In five cases, we identified a strong link (class A), in which ES categories were used as such to classify the criteria in the hierarchy. At the other end of the scale were five cases in which ESs were only mentioned in the text and the analyses were not explicitly linked to ES (class G). The rest of the cases were grouped between these.

**Table 1:** Classification of the papers according to the level of linkage to ESs

Class	Description	Cases	Link to ES concept
**A**	ES categories are used as such to classify the criteria in the hierarchy	Bryan et al. (2010); Karjalainen et al. (2013a, Case B)^a^; Liu et al. (2013); Esse et al. (2019); Saarikoski et al. (2019)	
**B**	ESs and their three/four categories form one branch in the hierarchy	McInnes et al. (2016); Kuller et al. (2019)	
**C**	ES categories are not presented in the hierarchy, but the criteria describing ESs are explicitly identified and highlighted.	de Jalon et al. (2014); Liquete et al. (2016); Maydana et al. (2020)	
**D**	The term ES is mentioned as an overarching goal of the analysis	Zhu et al. (2015); Odgaard et al. (2017); Comin et al. (2018); Borsuk et al. (2019)	
**E**	The term ES is used as an umbrella concept (title of branch) for some criteria	Bryan and Kandulu (2011); Johnston et al. (2013); Canada and Mariottoni (2016)	
**F**	ES is used as one aggregate criterion in the framework	Miller and Belton (2014)	
**G**	Neither the term ES nor any of the ES categories or classes are explicitly mentioned in the hierarchy or in the names of criteria, but the analysis is reported to deal with ESs in general	Karjalainen et al. (2013a, Case A); Karjalainen et al. (2013b); Hoenke et al. (2014); Huang et al. (2015); Roy et al. (2018); Beardmore et al. (2019)	

^a^In Karjalainen et al. (2013a), the same case was analyzed with both MCDA and ES concept (named as cases A and B, respectively)

We noticed that there was a rather small difference in the number of the lowest-level criteria between the cases that had a strong ES link (classes A and B in Table 1, 16 criteria on average), and cases that had weaker link (classes C–G, 13 criteria). For example, in two of the articles in class A, namely Liu et al. (2013) and Saarikoski et al. (2019), there were four ES categories but only eight lowest-level criteria.

Case-by-case needs are, naturally, crucial when deciding which ES are included in the analysis. For example, Comin et al. (2018) used 11 ES criteria in the multi-scale spatial analysis of ES in the River Piedra watershed to prioritize sites for ecological restoration. They excluded provisioning services because these are often not the goal of ecological restoration.

In the next phase, we analyzed the hierarchies to determine how the ES categories had been used in the case studies of the reviewed articles. Another interest was in identifying which non-ES criteria or aspects were used in the cases.

The ES concept covers different types of benefits of nature to people. In less than half of the cases (10/23), an ES category or class explicitly mentioned in the ES classification had been used as a criterion in the decision hierarchy (classes A–C in Table 1). However, without knowing the needs or goals of stakeholders, it is impossible to assess whether the hierarchies had been sufficiently comprehensive.

Interestingly, Karjalainen et al. (2013a) compared the ex post ES criteria developed by the research team with the primary MCDA hierarchy created with stakeholders. They identified two major differences in the set of criteria. First, in MCDA, supporting services, such as nutrient cycling and food webs, were not included, as the focus was on the socioeconomic issues emphasized by the stakeholders. Besides, the link between the restoration alternatives and supporting services was unclear. Second, when using the ES concept, disservices such as social and economic losses due to losses to hydropower generation, for example, would be excluded from the analysis. This reflection clearly shows that the decision on whether to use the ES concept can have a significant effect on the criteria.

Table 2 presents the ES criteria used in the articles having the strongest link to the ES concept. These not only include the articles using only ES criteria, but also articles in which some non-ES criteria were included to cover other aspects, such as technical, socio-economic, risk, and feasibility ones. Of the latter articles, Kuller et al. (2019) had 10 ES criteria and 18 non-ES criteria in the hierarchy, Saarikoski et al. (2019) included 9 ES criteria and 3 non-ES criteria, and McInnes et al. (2016) 15 non-ES criteria alongside 4 ES categories.

**Table 2:** Examples of cases having the strongest links to the ES concept (Classes A and B in Table 1). PS denotes provisioning services, RS regulation services, CS cultural services, and SS supporting services

Authors	Case and criteria used in the analysis
Bryan et al. 2010	Strategic management priorities for a river basin (ES hierarchy based on MEA)
	PS: Food and Fiber, Biochemical resources, Fresh water, Geological resources, Energy, Air quality, Climate
	RS: Air quality, Climate, Water quantity, Erosion, Water quality, Disease, pests and natural hazards, Pollination
	CS: Cultural diversity and heritage, Spiritual, sense of place and lifestyle, Knowledge and education, Aesthetics and inspiration, Social relations, Recreation and tourism, Bequest, intrinsic and existence
	SS: Soil formation, Photosynthesis and plant primary production, Nutrient cycling, Water cycling
Esse et al. 2019	Identification of ES in river catchments (ES hierarchy based on CICES V4.3)
	PS: Rural drinking water, Urban drinking water, Artisanal fishing, Industrial fishing, Grazing (livestock), Flora, Fauna, Seed production
	RS: Elimination of dilution with non-organic pollutants, Erosion regulation, CO2 sequestration, Surface run-off control
	CS: Thermal centers, Tourism, Natural beauty, Fishing, Science and research
Karjalainen et al. 2013a (Case B)	Evaluation of river restoration alternatives (ES hierarchy based on MEA)
	PS: Commercial harvest, Subsistence harvest
	CS: Local identity and amenity values, Tourism and attractiveness of the region, Recreational value/recreational fishing
	SS: Nutrient cycling, Sediment turnover, Aquatic/terrestrial food webs, River mussels
Kuller et al. 2019	Spatial suitability assessment of green urban stormwater infrastructure (‘Needs’ branch based on MAE)
	Opportunities: Biophysical (5 sub-criteria), Socio-economic (3), Planning and governance (10)
	Needs: Provisional (1 sub-criterion), Cultural (4 sub-criteria) and Regulating services (5 sub-criteria)
Liu et al. 2013	Water resources management - evaluation of ES values of sub-catchments (ES hierarchy based on TEEB)
	PS: Food and Fiber, Freshwater
	RS: Carbon sequestration, Moderation of extreme events
	CS: Spiritual and sense of place, Recreational and mental health, Esthetic, appreciation and cultural inspiration
	Habitat services: Carbon sequestration, Moderation of extreme events
McInnes et al. 2016	Evaluation of water quality improvement and ecosystem service provision (‘ES benefits’ branch based on MEA)
	Non-ES benefits: Buyer and Seller benefits (2 sub-criteria in both), Stakeholder benefits (2), Regulatory risks (4), Project costs (3), Technical effectiveness (2)
	ES benefits: Four ES categories (optimizing provisioning, regulating, cultural and supporting services). No criteria.
Saarikoski et al. 2019	Evaluation of peat extraction alternatives (ES hierarchy based on CICES V4.3 and enriched with socio-economic factors)
	PS: Energy peat, Horticultural peat, Berries
	RS: Carbon sequestration, Water quality, Biodiversity
	CS: Recreation, Landscape, Education
	Socio-economic factors: Employment, Regional Economy, Landowners’ freedom of choice

In three cases that utilized some ES classification system as such (Liu et al. 2013; Saarikoski et al. 2019) or systematically utilized the ES concept in identifying criteria (Karjalainen et al. 2013a, case B), the number of criteria was relatively low (8 or 9). According to Liu et al. (2013), one reason for the low number of ES criteria could be the lack of research data. On the other hand, the low effect or importance of an ES criterion may explain its absence (e.g., Karjalainen et al. 2013a).

One function of the ES concept is to raise awareness of the multiple ways that human well-being depends on well-functioning ecosystems (see, e.g., Ainschough 2019). To examine how awareness raising was considered in the cases, we performed a search of the 23 studied articles using the word “awareness”. Only two articles stated that the case/methods helped to raise awareness (Liquete et al. 2016; Comin et al. 2018). In several cases (Bryan and Kandalu 2011; Johnston et al. 2013; Huang et al. 2015), awareness raising was one of the management options, and in Kuller et al. (2019), environmental awareness was a socio-economic criterion in the spatial suitability assessment of green urban stormwater infrastructure.

### Consideration of Subjectivity: how was Preference Information Collected and Presented?

We classified the 23 cases into four classes in terms of how stakeholder preferences were considered in the analyses and the presentation of results (Table 3). First, there were five cases in which stakeholders were not engaged, and the weights were assigned by the authors of the article or a research team. In some of these cases (Miller and Belton 2014; Beardmore et al. 2019), sensitivity analysis was carried out to illustrate how changes in the criteria weights influenced the outcomes. Second, there were four cases in which opinions were collected from stakeholders but the weights were assigned by the authors based on the collected information. The preference information was presented most explicitly in seven cases in which preference information was collected from stakeholders mainly through personal interviews. Furthermore, the results were presented either with the profiles of individual interviewees or with a few divergent perspectives on stakeholders’ preferences.

**Table 3:** Presentation of preference information in the cases^a^

Class	Classification of the cases	Cases	Consideration of stakeholders’ subjective views
A	Stakeholder preference information was not collected; the authors assigned the weights (illustrative examples of methods)	Miller and Belton (2014), Canada and Mariottoni (2016), Roy et al. (2018), Beardmore et al. (2019), Kuller et al. (2019)	
B	Expert-generated preference/weight profiles on the basis of collected information	Hoenke et al. (2014), McInnes et al. (2016), Odgaard et al. (2017), Maydana et al. (2020)	
C	Preference information was collected from stakeholders. Differences in the preferences were not presented in the article	Bryan and Kandulu (2011), Liu et al. (2013), Huang et al. (2015), Zhu et al. (2015), Borsuk et al. (2019), Esse et al. (2019)	
D	Preference information was collected and preference profiles of selected stakeholder representatives or individual stakeholders were presented or stakeholder representatives were clustered from different perspectives	Bryan et al. (2010), Karjalainen et al. (2013a, 2013b), de Jalon et al. (2014), Liquete et al. (2016), Comin et al. (2018), Saarikoski et al. (2019)	

^a^Johnston et al. (2013) was not included, as it summarizes 7 cases and the results of the cases are not described in detail

Many different techniques were used to collect preference information from stakeholders. In several cases, weights were determined through interviews with stakeholder representatives (e.g., Karjalainen et al. 2013a, 2013b; Liquete et al. 2016; Saarikoski et al. 2019). Workshops (Borsuk et al. 2019) and online surveys (Liu et al. 2013) were also utilized. In some cases, information was collected by multiple methods; for example, Maydana et al. (2020) used the preferences from surveys, monitoring, expert judgments and key actor interviews. Not all cases applied an elicitation approach. For example, Hoenke et al. (2014) discussed criteria ranks with a group of stakeholders, and hence reflected professional and practitioner knowledge. A formal elicitation process in this case was expected to be too resource intensive given the large number of criteria and indicators.

In many cases (Group D, in Table 3), respondents assigned their weights individually, and the results were presented either individually or in a cluster of opinions. In three cases, weights were elicited in a deliberative discourse aiming at consensus weights. In the Myponga catchment case (Bryan and Kandulu 2011), the aim of the community forum (14 participants) was to arrive at a consensus view of the best policy alternative to address water quality objectives. In the iterative process, criteria weights were repeatedly revised as new information came to hand through discussion between group members and through interactions with experts until consensus was reached. In the Merrimick River watershed case (Borsuk et al. 2019), 11 panels of citizens (67 participants) evaluated 10 ESs in four full-day workshops. After group deliberation and expert scientific input, all groups except one were able to reach an internal consensus on the relative value of the ESs.

Saarikoski et al. (2019) also applied deliberative valuation workshops (citizen juries) in a case related to peat extraction in Finland. In this case, three sets of 9–11 participants took part in a series of three workshops (for details, see Saarikoski and Mustajoki 2021). The participants were asked to fill in a questionnaire about their opinions of the importance of different ESs both before and after the whole process. For some ESs, there were differences in the preferences of the stakeholders were smaller after the deliberative process than before, but no consensus on the desirable use of peatlands was reached.

Mavrommati et al. (2017) describe a carefully designed and interesting elicitation process used in the Merrimack River watershed case (Borsuk et al. 2019). They realized the weight elicitation process by using the swing technique (von Winterfeldt and Edwards 1986) with position cards on which the differences in scenarios’ impacts on criteria were made visible to the participants, and participants were asked to place the cards on a scale of 0–100.

### Advantages and Pitfalls of the Complementary use of the ES Concept and MCDA in Water Management

The advantages of the complementary use of the ES concept and MCDA can be assessed either by analyzing the added value of utilizing the ES concept in an MCDA process or vice versa. In practice, all the MCDA cases in water management dealt with some ESs, even if the ES concept or terminology was not used in these articles. Thus, our focus here is on examining the additional value of explicitly using the ES concept. Based on the findings of the reviewed case studies, Table 4 presents the additional values—and also the pitfalls—of both of these approaches.

**Table 4:** Potential additional value and pitfalls for both the MCDA-driven process utilizing the ES concept and for the ES-driven process utilizing MCDA based on the 23 analyzed articles

Additional value of utilizing the ES concept within MCDA	Pitfalls of utilizing the ES concept in MCDA
- Provides a systematic framework and checklist for identifying the criteria and targeting further ESs (Odgaard et al. 2017; Saarikoski et al. 2019)	- May restrict innovative thinking and conceptual diversity in structuring the problem (Karjalainen et al. 2013a; Saarikoski et al. 2019)
- Promotes recognition of the value provided by ecosystems (e.g., Liquete et al. 2016; Saarikoski et al. 2019)	- May increase the risk of double-counting (Liu et al. 2013; Liquete et al. 2016)
- Helps in providing performance metrics for monitoring the state of ESs (Miller and Belton 2014)	- The ES terminology may be complex and distant to stakeholders (Karjalainen et al. 2013a)
- Provides an integrated approach to incorporate ESs into environmental analysis with rigid links between ecosystem characteristics and benefits for people (Karjalainen et al. 2013a)	- In the case of a large variety of ESs, it may be difficult to identify simple descriptors of performance (Miller and Belton 2014)

Additional value of utilizing MCDA in ES analysis	Pitfalls of utilizing MCDA in ES analysis
- Facilitates the identification of criteria (McInnes et al. 2016) and supports a wider perspective in the inclusion of criteria (Karjalainen et al. 2013a)	- Depending on how MCDA is carried out, it may open up rather than close down the policy discourse (Karjalainen et al. 2013b)
- Applying value-focused thinking during the scoping stage enables the identification of priority ESs (Karjalainen et al. 2013a)	- The additivity assumption, which is made, for instance, in MAVT, does not necessarily apply (Borsuk et al. 2019)
- Forms a structured and shared framework for making trade-offs (McInnes et al. 2016, Roy et al. 2018)	- Determining the weights of the criteria is prone to different types of biases (Kuller et al. 2019)
- Supports open and transparent weighting of criteria, which promotes consensus and accountability in the final outcomes (McInnes et al. 2016; Huang et al. 2015).	- Making sense of multiple, diverse stakeholder priorities requires particular consideration (Bryan et al. 2010)
- Provides structure and transparency for linking stakeholder engagement to the complex decision-making process (Bryan et al. 2010; Bryan and Kandulu 2011; Liu et al. 2013; Borsuk et al. 2019) and facilitates learning (Saarikoski et al. 2019).	

## Discussion

### General Findings from the Combined use of ES Concept and MCDA

The analysis of 24 cases showed that the ES concept and MCDA can be combined in many different ways. There were, for example, large differences in what ES categories and criteria were used in MCDA. MEA was used as a framework in most of the older cases, but the CICES was also used especially among the newest cases (e.g., Comin et al. 2018; Esse et al. 2019; Saarikoski et al. 2019). From the terminology used in several decision hierarchies it can, however, be inferred that the ES concept has been utilized in the background, even if their upper level categories were not used.

The analysis also indicates that the use of the ES concept has not precluded the use of other criteria if this has been necessary for the case. This is supported by the fact that non-ES criteria were also considered in those cases in which the link to the ES concept was strongest. The cases also suggest that the use of the ES concept does not necessarily lead to larger hierarchies, even if some previous literature suggests a risk of this (e.g., Mustajoki et al. 2020).

The range of weighting processes in the cases varied greatly, underlining that there is no specific protocol for weighting in MCDA, but the used approach depends on the methodological expertise of the analysts, the participants’ skills and time resources, as well as case-specific needs. On the one hand, it allows case-specific characteristics to be taken into account, but on the other hand it can also lead to processes that do not follow MCDA theory. In these cases, there is a risk that weights are distorted by various procedural and cognitive biases (e.g., Montibeller and von Winterfeldt 2015) and do not necessarily correspond to the actual preferences of participants. When designing the weight elicitation process it is important that the manner the weights are used are taken into account (Choo et al. 1999).

There is no unambiguously best MCDA method for assessing ESs, as each method has its strengths and weaknesses, and no single method would be best in all decision situations (Hobbs and Horn 1997, Guitouni and Martel 1998). The way the method is applied is often more relevant than the choice of method. An exception, however, is a situation where the requirement is strong sustainability (Borsuk et al. 2019), which does not allow trade-offs to be made between all ES categories. In such situations, non-compensatory methods such as ELECTRE or PROMETHEE are appropriate. In cases with weak sustainability, trade-offs are acceptable, and compensatory methods such as MAVT and AHP can be used.

### Additional Value and Pitfalls of Utilizing the ES Concept in the MCDA Process

The additional value of utilizing the ES concept in MCDA in addressing water management is mostly related to the systematic framework and classification systems for identifying ESs relevant to the problem and provided by the ES concept (Table 4). Identifying a comprehensive set of ESs that are relevant to the given planning or decision-making situation is certainly beneficial for MCDA (see also Odgaard et al. 2017). Furthermore, this may promote recognition of the value provided by ecosystems (Liquete et al. 2016) and the broadening of perspectives on potential issues in linking ecosystem properties to human benefits and values (Karjalainen et al. 2013a; see also Mustajoki et al. 2020). Karjalainen et al. (2013a) also stressed that the explicit consideration of ESs within the MCDA framework would have enabled the framing and valuing of some provisioning services (commercial and subsistence harvesting of salmon) in a more meaningful way for some stakeholders.

The use of the ES concept can also increase the comparability of the cases (Finisdore et al. 2020) by providing a common set of criteria (Liu et al. 2013). This is especially important in situations where a common indicator system is created (e.g., Liu et al. 2013; Zhu et al. 2015; Odgaard et al. 2017). Besides common criteria, the ES classification system can additionally help in providing common performance metrics for monitoring the state of individual ESs (Miller and Belton 2014). Karjalainen et al. (2013a) also concluded that if more ecological expertise is used when applying the ES concept, the whole process of selecting the key ESs and the assessment criteria would be more expert-driven and would better consider ecosystem characteristics, such as the relationship between mussels and salmon management. However, the ES concept can also be used to include the detailed identification of losers and beneficiaries and where they are located (Karjalainen et al. 2013a, see also Turner et al. 2010). Highly illustrative heatmaps, which use color classification to describe, for example, the value of ESs in specific areas, can be used to present the results of ES analyses (e.g., Comin et al. 2018; Odgaard et al. 2017; Kuller et al. 2019).

Many of the examples of additional value and pitfalls listed in Table 4 strongly depend on the implementation of the process and are in fact opposite sides of the same coin. For example, using ES classification to ensure the inclusion of all the relevant aspects in the evaluation can reduce stakeholder learning and commitment to the process. This may especially be the case if it is used too early without allowing the stakeholders to first bring up their thoughts (Saarikoski et al. 2019). This may also limit creative thinking regarding issues that would need to be considered and that are not adequately captured by the ES concept (e.g., Karjalainen et al. 2013a, 2013b, Saarikoski et al. 2019). Furthermore, one should also pay attention to communication of the ES concept to the stakeholders, as excessively rigid use of ES concept vocabulary may reduce the intuitiveness of the discussed issues. For more detailed discussion see, for example, Díaz et al. (2018), Primmer et al. (2018), Ainscough et al. (2019) and Flood et al. (2020).

Baker et al. (2013) concluded that the ES concept provides a potentially valuable framework for environmental assessment, but context-specific consideration is needed to assess whether it can provide added value. In those cases that require absolute monetary values for ESs, other approaches, such as CBA (Saarikoski et al. 2016), often supported by economic (stated/revealed) valuation methods, should be used toward the end of the evaluation process.

Although double-counting has been identified as a potential problem in the use of the ES concept (e.g., Hein et al. 2006; Turner et al. 2010; Nahlik et al. 2012), it was only mentioned in five of the reviewed articles (Borsuk et al. 2019; Bryan et al. 2010; Liquete et al. 2016; Liu et al. 2013, Miller and Belton 2014). It may be that this challenge has already been partially resolved, as the articles that mentioned this problem were clearly older than those articles presently evaluated in our review (see also Finisdore et al. 2020, 2021). On the other hand, the use of the ES concept may also simplify the assessment too much, as important aspects can be excluded from the analysis. For example, Liu et al. (2013) noted that analysis based on the valuation of certain ESs and single indicators under these may have oversimplified the analysis of a complex system.

### Additional Value and Pitfalls of Utilizing MCDA in the ES Evaluation Process

Building the evaluation framework is a crucial step in any planning process, and MCDA can provide significant added value to this phase (Karjalainen et al. 2013a; McInnes et al. 2016). Experiences from the analyzed cases suggest that MCDA can be useful in the evaluation of ESs by providing a framework for comparing alternatives and utilizing stakeholder preference information (i.e., the final steps of the process in Fig. 1; see, e.g., McInnes et al. 2016; Roy et al. 2018).

According to McInnes et al. (2016) and Roy et al. (2018), considering the trade-offs required for achieving benefits from ESs and goods can benefit from using the MCDA approach. According to Karjalainen et al. (2013a), including negative economic impacts (e.g., losses in hydropower production) in the analysis lowers the risk that economically inefficient and, for some groups, unacceptable alternatives are preferred. MCDA can also be used to highlight issues for which there is agreement and disagreement (McInnes et al. 2016, Canada and Mariottoni 2016). These may concern the weights of the criteria, the desirability of the alternatives for the different criteria (estimates may be subjective, e.g., landscape, or there may be different views on the magnitude of the impacts between the alternatives), and the outcome of the analysis (i.e., the ranking and relative desirability of the alternatives). However, some values, such as economic costs or “disbenefits”, might not naturally fit into the ES concept, and it is important that these are not dismissed as hidden externalities but are also addressed (Karjalainen et al. 2013a).

How useful and beneficial the ES and MCDA approaches are in terms of individual and social learning depends greatly on the way in which they are implemented. Central to this is the timing of stakeholder involvement and its intensity at different stages of the process. Among the analyzed cases there were several where the interaction between different stakeholders was close from the structuring stage of the problem (e.g., Karjalainen et al. 2013a; Borsuk et al. 2019). Deliberative weight elicitation (Proctor and Drechsler 2006) based on a discursive process was only realized in three cases (Bryan and Kandulu 2011; Borsuk et al. 2019, Saarikoski et al. 2019).

Borsuk et al. (2019) found that the deliberative process influenced the stakeholders’ preferences, and that the group preferences reflect a consensus, rather than simply the tallied preferences of the majority (Borsuk et al. 2019). Although the integration of larger groups might be difficult under deliberative approaches (Langemayer et al. 2016), it can be worthwhile to develop more carefully considered, and thus more robust weights. In addition, the deliberative process can further enhance the depth and breadth of understanding of the issues. Bryan and Kandulu (2011) found this level of understanding critical for tailoring an effective policy mix and sequence in this case study and for effective design of agricultural non-point source pollution policy in other catchments.

MCDA can also provide a means for analyzing uncertainties by sensitivity analysis. In the most non-spatial articles of our study comparing policy options, sensitivity analyses for criterion weights were performed. Different methods have been applied in sensitivity analysis, such as Monte Carlo simulation (e.g., Bryan et al. 2010; McInnes et al. 2016), deviation of the weights of the criteria or the values of the alternatives (e.g., Liquete et al. 2016; Beardmore et al. 2019), and various combinations of weights on different weight profiles (e.g., Liu et al. 2013; Maydana et al. 2020). In contrast, in GIS-MCDA applications, which presented the results in maps, sensitivity analysis was not a common practice.

There was little discussion about the limitations of MCDA regarding the valuation of ESs in the reviewed articles. One exception is Kuller et al. (2019), who described on a general level the existence of different types of weighting biases, and stated that due to the nature of GIS-MCDA, a certain level of uncertainty and bias is unavoidable. Therefore, they conclude, clear documentation of the choices made is crucial, and clear user guidance is necessary. In MCDA, a strong assumption regarding additivity and preference independence is typically employed for simplicity. However, there are situations where these assumptions may be violated (Borsuk et al. 2019). Other potential limitations include the inability to deal with multiple scales of ES supply and demand (Langemayer et al. 2016) and the risk of opening up rather than closing down the policy discourse (Stirling 2006). However, one should note that opening up the case can sometimes also be very useful, especially in the early phases of the process (Karjalainen et al. 2013a). On the other hand, aspects that are only partially or indirectly based on ESs may be omitted in the analysis (e.g., heritage/historic environment, aspects of health; Baker et al. 2013).

### Recommendations Regarding the Complementary use of MCDA and the ES Concept

In the analysis of the 23 articles, we identified the following good practices that could be of wider benefit in applying the MCDA and the ES concept in a complementary way:

- Choice of the methods: When selecting what MCDA methods to use, carefully consider the needs of the case and make the selection of ES categories on the basis of this. For example, in cases of strong sustainability, non-compensatory methods such as ELECTRE are most appropriate (e.g. Borsuk et al. 2019), whereas compensatory methods such as MAVT can be used when trade-offs between ES categories are allowed.
- Stakeholder involvement: Plan the intensity of the stakeholder involvement process according to the purpose of the analysis. Cases aimed at raising awareness do not need such close stakeholder involvement (e.g. Beardmore et al. 2019), but for learning by analysing, close collaboration with the stakeholders is required (e.g. Karjalainen et al. 2013a).
- Criteria selection. Use the ES concept at least as a checklist when creating the MCDA hierarchy in water management cases. At best, the selection of criteria is based on both the innovative thinking stemming from the principles of value-focused MCDA (Keeney 1992) and the utilization of the ES concept for covering all the different ESs.
- Stakeholders’ preferences. Utilize and make transparent the use of stakeholder and expert preferences in the evaluation. It is a good practice to present the details of the weight elicitation protocol (see, e.g., Liquete et al. 2016). Visits by respondents to the study area before determining the weights of the criteria can be useful (Comin et al. 2018).
- Visualization. Visualize the results and present a wide spectrum of preferences to highlight the importance of subjectivity (e.g., Liquete et al. 2016; Saarikoski et al. 2019). Also, make the criteria based on ES categories visible in the MCDA hierarchy. See de Jalon et al. (2014) for an example of highlighting these in the hierarchy to differentiate them from the socioeconomic criteria.
- Understandability. Design a concrete, understandable, and theoretically valid weight elicitation procedure to avoid cognitively too demanding or meaningless questions and biased results (for a good example, see Mavrommati et al. 2017). Use terminology that is familiar to the stakeholders, as some terms related to ESs can be abstract or can have strong meanings for people.
- Evaluation of the process. Carefully report the process, and collect, analyze and report the experiences from participants involved in the evaluation. Reflection on experiences can also benefit other researchers and practitioners in designing how to apply the method and improve MCDA practices in the long term. For a good example of reporting the process, see Borsuk et al. (2019), and for the participants’ experiences, see Karjalainen et al. (2013b).

What can be considered a good practice also depends on the characteristics and constraints of the case. For example, regarding how stakeholders were engaged and how the subjectivity was considered in the analysis, we identified three significantly different approaches, each of which can be considered a good practice in certain situations (see Table 3, Classes A, B, and D).

## Conclusions

In this paper, we have analysed 23 articles to examine the complementary use of MCDA and ES concept in water management. In general, there was no single specific way to combine the approaches, but their application varied greatly. A key question in the structuring phase is in which stage the ES concept should be included in the discussion. We suggest that stakeholders first have a chance to articulate their goals and objectives without any strict framework, as it can restrict innovative thinking. The ES concept can later be used as a checklist to ensure that nothing essential has been omitted from the analysis.

Based on our analysis, water management decisions typically deal with many crucial criteria that are not included in the ES concept, such as employment, the regional economy, the feasibility of alternatives, and risks associated with them. On the other hand, ES terminology can be challenging for laymen and thus affect the applicability of the ES concept in the case. Nevertheless, if the ES classification is used as such or included as a part of the MCDA hierarchy, careful attention should be paid to designing the weight elicitation process and trade-off questions such in a way that they are theoretically valid and not cognitively too demanding for the participants. For example, the comparison of three ES categories (provisional, cultural, regulating) at the top level of the hierarchy can be cognitively demanding and lead to distorted weights without well-thought-out questions and guidance from the analyst.

Profound knowledge of the strengths and challenges of the MCDA and the ES concept is the only way to achieve the full benefits of the complementary application of the methods. However, the need to involve different stakeholders and their intended role may have an impact on which type of approach should be chosen. The active role of stakeholders in many cases indicates, on the one hand, that MCDA is a very suitable tool to facilitate multi-stakeholder processes, and on the other, that MCDA needs input from stakeholders, as preferences and criteria weights are subjective.

There is still a lack of experiences of the joint use of the approaches, and we encourage other researchers to continue with the topic. Topics for further research include the development and wider adoption of good practices for weight elicitation (Mavrommati et al. 2017 is a good example), the development of weighting procedures for different types of cases, analysis of what types of indicators have been used in the evaluations (monetary, non-monetary), and analysis of how different types of indicators should be considered in the weighting of the criteria. GIS-based cases, where there is a need to evaluate different geographical areas (e.g. areas that should be targeted for remediation measures), differ greatly from those that assess and evaluate alternative ways to realize remediation. Also, conducting similar types of reviews as this on other topics with a larger number of available cases could bring new insights. It would also be worth implementing practical applications so that the strengths and weaknesses of both methods are recognized in the design phase of the process, and the experiences gained are carefully evaluated and reported.

## Supplementary information

Supplementary information
